# Inflammatory myofibroblastic tumor of the transverse colon with synchronous gastrointestinal stromal tumor in a patient with ulcerative colitis: a case report

**DOI:** 10.1016/j.ijscr.2019.06.012

**Published:** 2019-06-12

**Authors:** Eugenia Raffaeli, Luca Cardinali, Maurizio Fianchini, Donatella Brancorsini, Piergiorgio Mosca, Cristina Marmorale

**Affiliations:** a Department of General Surgery, Polytechnic University of Marche - Azienda Ospedaliero-Universitaria Ospedali Riuniti “Umberto I-G.M. Lancisi-G. Salesi”, Ancona, Italy; b Department of Experimental and Clinical Medicine, Section of Surgical Sciences, Polytechnic University of Marche, Ancona, Italy; c Section of Pathological Anatomy and Histopathology, Università Politecnica delle Marche, Azienda Ospedaliero-Universitaria Ospedali Riuniti “Umberto I-G.M. Lancisi-G. Salesi”, Ancona, Italy; d Digestive System Diseases, Endoscopy and Inflammatory Bowel Diseases Unit, Ospedali Riuniti, Ancona, Italy

**Keywords:** Inflammatory myofibroblastic tumor, IMT, Ulcerative colitis, Gastrointestinal stromal tumor, GIST, Inflammatory pseudotumor

## Abstract

**Introduction:**

Inflammatory myofibroblastic tumor (IMT) is a rare proliferative disease of uncertain etiology, characterized by the proliferation of fusate or epithelioid myofibroblasts admixed with predominantly mononuclear inflammatory cells. IMT is generally considered a benign lesion, although in some cases this neoplasm has shown an aggressive behavior in terms of local recurrence and metastasis. We report the case of a patient with a ten-year history of ulcerative colitis affected by IMT of the transverse colon and by synchronous gastrointestinal stromal tumor (GIST) of stomach.

**Presentation of case:**

A 59-year-old woman with a ten-year history of ulcerative colitis has been admitted to our hospital with signs and symptoms of acute recurrence of ulcerative colitis: abdominal pain, diarrhea, hematochezia and rectal tenesmus. Colonoscopy showed a left colon with diffuse hyperemia, mucosal erosions and a 2-cm, irregularly shaped, polypoid lesion at the level of the transverse colon. Histopathological examination of the specimen obtained via biopsy of the polypoid lesion has revealed a mesenchymal neoplasm with uncertain characters of malignancy. Due to the severity of the inflammatory bowel disease resistant to immunosuppressive and steroid drug treatment, surgical indication was given.

**Discussion:**

Although the relationship between IMT and Crohn’s disease has been widely reported in literature, the relationship between IMT and ulcerative colitis has never been previously described.

**Conclusion:**

To the best of our knowledge, this is the first case of IMT associated with ulcerative colitis reported in literature and the synchronous association with a gastric GIST represents another primacy.

## Highlights

• Inflammatory myofibroblastic tumor (IMT) is a rare pathology with uncertain etiology.
• Patient with ulcerative colitis affected by synchronous colic IMT and gastric GIST.
• In our case, IMT mimicked an adenocarcinoma of colon on an ulcerative colitis (UC).
• The relationship between IMT and UC has never been previously described.
• The synchronous association between IMT of colon and gastric GIST is another primacy.

## 1 Introduction

Inflammatory myofibroblastic tumor (IMT), also known as inflammatory pseudotumor (IPT) or plasma cell granuloma (PCG), is a rare proliferative disease of uncertain etiology, firstly described in 1937 as a primary pulmonary lesion [1]. Clinical manifestations widely vary depending on the anatomic site and can include indurated mass or swelling, fever, weight loss, pain and specific symptoms related to the site of origin. IMT mostly affects children and young adults, while being observed in people of any age, and generally does not exhibit any gender preference [1]. Two age peaks in the incidence of IMT have been reported in a recent review: one in pediatric age and the other between 50 and 60 years of age [2]. The most frequent site of IMT is the liver followed, in descending order, by the lung, the head-neck district, the abdomen and urogenital system [2].

Microscopically, the IMT consists in the proliferation of fusate or epithelioid myofibroblasts admixed with predominantly mononuclear inflammatory cells [1]. The diagnosis of IMT is based on the immunohistochemistry study showing tumor cells as characteristically positive for smooth muscle actin (SMA) with or without desmin expression; focally positive for vimentin and negative for CD117 and CD34 [1,3]. IMT is generally considered a benign lesion, although in some cases this neoplasm has shown an aggressive behavior in terms of local recurrence (20% of cases) and metastasis (most rarely) [1]. Most recent studies have shown that the prognosis of the IMT depends on the rearrangements of the anaplastic lymphoma kinase (ALK) gene, locus on chromosome 2p23, causing aberrant ALK expression [4]. Rearrangements involving the ALK gene have been documented in approximately 50% of IMTs and they constitutively active ALK expression [5]. Several ALK fusion proteins, including TPM3-ALK found in IMT, induce transformation in cell lines and animal models [6], a finding that suggests that ALK rearrangement may define a subgroup of IMTs that is sensitive to targeted kinase inhibition. Distant metastases occur primarily in ALK-negative IMTs, but local recurrence occurs regardless of ALK expression [3]. IMTs with ALK immunoreactivity on the nuclear membrane or in the peri-nuclear site could have a more favorable prognosis with a lower risk of relapse, and they could respond to crizotinib therapy [7].

Surgical resection is the treatment of choice [4]. To date, little evidence is available concerning the role of chemotherapy in the treatment of IMT, and most data concern pediatric population. Radiation therapy has reported some benefits in pulmonary IMT, while there is no significant evidence for extra-pulmonary sites [8].

We report a case of inflammatory myofibroblastic tumor in a patient with synchronous gastrointestinal stromal tumor (GIST) and ulcerative colitis.

This work has been reported in line with the SCARE criteria [9].

## 2 Presentation of the case

**Fig. 1:**
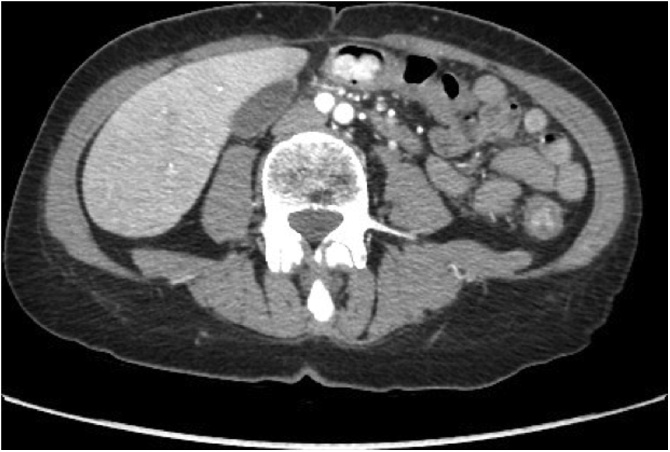
Computed tomography (CT) shows nodule formation of transverse colon with intense enhancement.

A 59-year-old woman with a ten-year history of ulcerative colitis, a previous episode of cytomegalovirus colitis and suffering from arterial hypertension and atrial fibrillation, has been admitted to our hospital with signs and symptoms of acute recurrence of ulcerative colitis: abdominal pain, diarrhea, hematochezia and rectal tenesmus. Laboratory analysis showed microcytic hypochromic anemia and neutrophilic leukocytosis. Colonoscopy showed a left colon with a tubulized appearance without haustra associated with diffuse hyperemia, mucosal erosions and a 2-cm, irregularly shaped, polypoid lesion at the level of the transverse colon. Histopathological examination of the specimen obtained via biopsy of the polypoid lesion has revealed a mesenchymal neoplasm with uncertain characters of malignancy. Contrast-enhanced computed tomography (CT) of the thorax and abdomen revealed an inhomogeneous hypodense nodule formation of 2.5 × 1.6 cm in diameter, with intense enhancement in the arterial phase localized to the middle-distal transverse colon (Fig. 1).

Due to the severity of the inflammatory bowel disease resistant to immunosuppressive and steroid drug treatment, surgical indication was given. It was therefore decided to proceed with a total colectomy with terminal ileostomy. The exploratory laparotomy revealed the presence of an unknown exophytic mass in the anterior stomach wall, which was removed. No postoperative complications were reported and the patient was discharged 7 days after surgery. In the following months, a proctectomy with ileoanal anastomosis (J-pouch) was performed. Ileostomy reversal was performed one month after the last surgery.

**Fig. 2:**
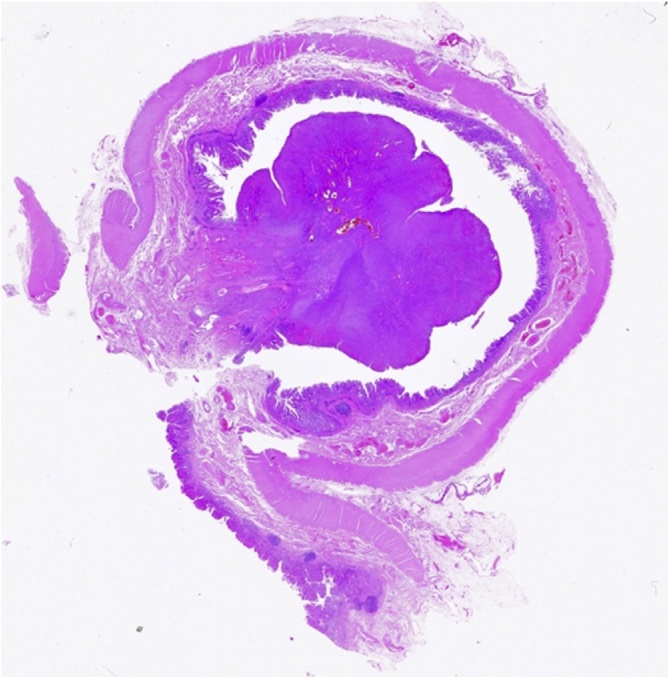
Photomicrograph of ulcerated polypoid lesion of transverse colon surrounded by a mucosa with increased consistency and velvety surface.

**Fig. 3:**
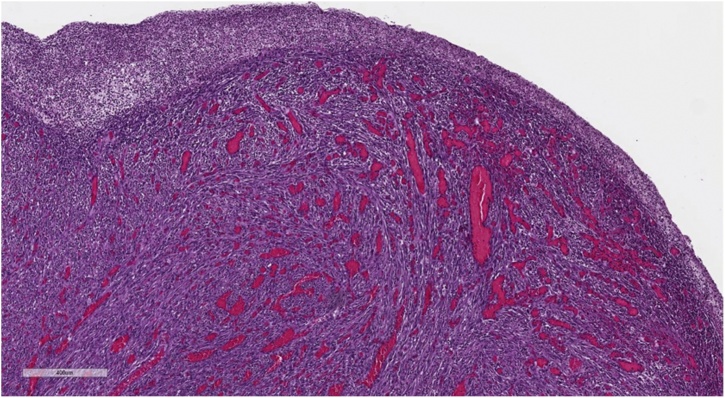
Photomicrograph of polypoid lesion of transverse colon shows a mesenchymal proliferation consisting of spindle cells organized in bundles.

**Fig. 4:**
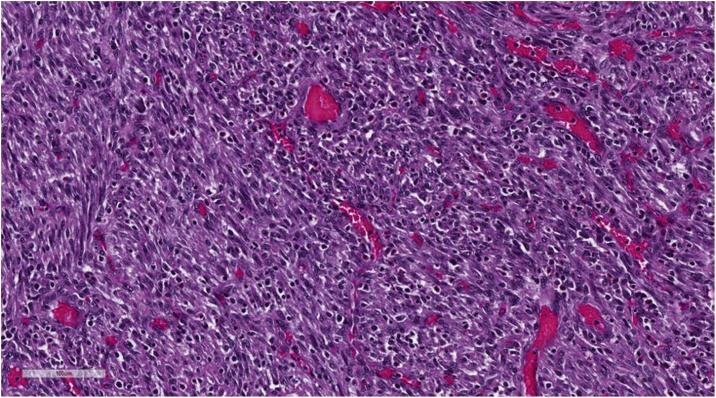
Photomicrograph of polypoid lesion of transverse colon shows a diffuse infiltration of lymphocytes, plasma cells and eosinophils associated with monomorphic spindle cells.

Histological examination of the colon revealed the presence of an ulcerated polypoid lesion of 2 × 2.8 cm in diameter surrounded by a mucosa with increased consistency and velvety surface extended for 3 cm (Fig. 2). Microscopic examination of the neoplasm showed a mesenchymal proliferation consisting of spindle cells, monomorphic and without significant atypia, organized in bundles and associated with diffuse infiltration of lymphocytes, plasma cells and eosinophils extending to the mucosa and infiltrating the submucosa (Fig. 3, Fig. 4).

The lesion was subjected to immunohistochemical staining which showed focal positivity for SMA, negativity for cytokeratin, desmin, S-100, Melan-A, CD34, CD117, DOG-1, CD21, CD23, CD1a and ALK. Based on these findings IMT diagnosis was confirmed.

Histopathological examination of the gastric lesion revealed a GIST.

A surveillance approach was decided, and the thoracic-abdominal CT scan performed 6 months after surgery showed no evidence of disease recurrence.

## 3 Discussion

Historically, the terms "inflammatory myofibroblastic tumor” (IMT), "inflammatory pseudotumor" (IPT) and "plasma cell granuloma" (PCG) have been used as synonyms or interchangeably. Nowadays the common use of the same name to identify these different entities has been criticized [10]. In fact, although being identifiable in a morphological-histopathological-molecular continuum, these lesions represent different entities [2]. IMT identify a heterogeneous group of single or multiple masses histologically characterized by fibroblastic or myofibroblastic proliferation with polymorphic, inflammatory infiltrates admixed with variable fibrosis, necrosis, and granulomatous reaction [10,11]. The precise etiology of IMT remains unclear. The association of IMT with infections by oncogenes virus, mutations on the ALK gene, DNA aneuploidy, clonal chromosomal abnormalities and their potentially aggressive clinical behavior – tendency of local recurrence with low risk of metastasis – [1] have prompted recent literature to designate a tumor etiology to these lesions [12].

According to a recent review reporting the most common sites of IMT, colon-rectum is the fifth abdominal site most commonly affected. It is preceded only by liver and biliary tract, spleen, peritoneum and stomach. Jejunum-ileum, duodenum, pancreas and esophagus were reported as less commonly affected sites [2]. The most frequently reported symptoms of colorectal IMT are intestinal irregularity, constipation, diarrhea, chronic hematochezia, intestinal occlusion, colo-colic or ileo-colic intussusception [4]. At imaging, colorectal IMT occurs as a non-capsulated, ill-defined, protruding or infiltrative intra-luminal mass. Prognosis of colon-rectum IMT seems to be more favorable than other sites. Based on the presence of metastases and relapses, in some cases of extracolonic IMTs (especially in omentum and mesentery site), an aggressive behavior cannot be excluded [1] and a long-term follow-up is recommended. IMT is currently regarded as a neoplasm of intermediate biologic potential because of its tendency to locally recur but rarely metastasize [1]. Therefore, a long-term follow-up is strongly recommended especially in case of IMT wider than 80 mm or locally advanced.

To the best of our knowledge, this is the first case of IMT of colon associated with synchronous ulcerative colitis and gastrointestinal stromal tumor (GIST) of stomach reported in literature.

To date, only five cases of IMT – three in liver, one in pancreas and one into the cerebellum - associated with Crohn’s disease have been reported [[13], [14], [15], [16]]. Although all patients with liver IMT showed an active chronic inflammatory bowel disease at hospitalization, it has been diagnosed promptly only in one case. Similarly, the diagnosis of inflammatory bowel disease was made belatedly (after 6 months) in the patient with pancreas IMT. These cases highlight that an underlying diagnosis of inflammatory bowel disease should be considered in patients with IMT. In our case, however, IMT mimicked an adenocarcinoma of colon on an ulcerative colitis.

Historically, the term gastrointestinal stromal tumor (GIST) identified a heterogeneous group of neoplasms of the gastrointestinal tract originating from the precursors of the interstitial cells of Cajal [17]. To date, GIST is defined as a specific, KIT-expressing and KIT-signaling driven mesenchymal tumor of the gastrointestinal tract [18]. IMT and GIST are classified as mesenchymal tumors and are both characterized by the presence of fusate cells. Nowadays, only two cases of GIST associated with hepatic IMT have been reported in literature [19,20]. In both cases, hepatic lesions were wrongly diagnosed as GIST metastases: only the histopathological study of the surgical piece allowed performing a proper diagnosis (rectum GIST with hepatic IMT and ileum GIST with liver IMT).

## 4 Conclusion

IMT is a rare pathology with uncertain etiology, on the edge between neoplasia and inflammatory proliferative reaction. Although the relationship between IMT and Crohn's disease has been widely reported in literature, the relationship between IMT and ulcerative colitis has never been previously described. To our knowledge, the synchronous association between IMT of colon and gastric GIST represents another primacy in literature.

The double uniqueness of association (IMT and RCU, IMT and GIST) referred to in this case-report makes it difficult to draw conclusions that can be useful in clinical practice. For this reason, comparison with other future similar case-reports are necessary.

## Declaration of Competing Interest

All authors have no conflicts of interest to declare.

## Sources of funding

This research did not receive any specific grant from funding agencies in the public, commercial, or not-for-profit sectors.

## Ethical approval

This study did not require ethical approval as it was exempt in our institution.

## Consent

Written informed consent was obtained from the patient for publication of this case report and accompanying images. A copy of the written consent is available for review by the Editor-in-Chief of this journal on request.

## Author contributions

**Raffaeli Eugenia:** Conceptualization, Writing – Original Draft, Writing – Review & Editing, Visualization. **Luca Cardinali:** Conceptualization, Writing – Original Draft, Writing – Review & Editing, Visualization. **Maurizio Fianchini:** Visualization. **Donatella Brancorsini:** Visualization. **Piergiorgio Mosca:** Visualization. **Cristina Marmorale:** Visualization, Supervision.

## Registration of research studies

This paper is a clinical report, no research involved.

## Guarantor

Cristina Marmorale.

## Provenance and peer review

Not commissioned, externally peer-reviewed.
